# Improving Gas-Sensing Performance Based on MOS Nanomaterials: A Review

**DOI:** 10.3390/ma14154263

**Published:** 2021-07-30

**Authors:** Shirui Xue, Sicheng Cao, Zhaoling Huang, Daoguo Yang, Guoqi Zhang

**Affiliations:** School of Mechanical and Electrical Engineering, Guilin University of Electronic Technology, Guilin 541000, China; shiruixue4268@163.com (S.X.); cao_sicheng@163.com (S.C.); G.Q.Zhang@tudelft.nl (G.Z.)

**Keywords:** MOS gas sensors, gas-sensing properties, improvement methods, gas-sensing mechanism, research ideas

## Abstract

In order to solve issues of air pollution, to monitor human health, and to promote agricultural production, gas sensors have been used widely. Metal oxide semiconductor (MOS) gas sensors have become an important area of research in the field of gas sensing due to their high sensitivity, quick response time, and short recovery time for NO_2_, CO_2_, acetone, etc. In our article, we mainly focus on the gas-sensing properties of MOS gas sensors and summarize the methods that are based on the interface effect of MOS materials and micro–nanostructures to improve their performance. These methods include noble metal modification, doping, and core-shell (C-S) nanostructure. Moreover, we also describe the mechanism of these methods to analyze the advantages and disadvantages of energy barrier modulation and electron transfer for gas adsorption. Finally, we put forward a variety of research ideas based on the above methods to improve the gas-sensing properties. Some perspectives for the development of MOS gas sensors are also discussed.

## 1. Introduction

In daily life, gas sensors have been used in various areas, including environmental monitoring, medical diagnosis, and agriculture [1,2,3,4,5]. In 1953, Brattain et al. [6] found the properties of semiconductors were affected by the change in the components of surrounding gases. In 1962, Seiyama et al. [7] manufactured the first metal oxide semiconductor-based gas sensor, which solved the problem of toxic gas adsorption and detection. With the development of advanced manufacturing technology and new materials, high-performance gas sensors based on different principles and structures have been widely developed [8,9]. Multiwalled carbon nanotubes and a graphene gas sensor have been successfully developed and manufactured by Dilonardo et al. [10] and Hayasaka et al. [11]. However, traditional nanomaterials sensitize the adsorption of toxic gases, accompanied by a decline in performance and the generation of by-products. In order to develop a stable and efficient gas sensor, the metal oxide semiconductor (MOS) has attracted researchers’ attention due to its excellent properties in gas sensing.

Gas sensors based on MOS materials have many advantages compared to others such as the fast response, low cost, and easy operation [12]. Shendage et al. [13] reported a WO_3_ thin-film sensor whose response was about 10 towards 5 ppm NO_2_ and about 131.75 towards 100 ppm NO_2_. Choi et al. [14] fabricated a SnO_2_ nanowire gas sensor. When the NO_2_ concentration was 0.5 and 5 ppm, its responses were 18 and 180 in 200 °C, respectively. However, there are some factors limiting its performance. The operation temperature of pristine MOS gas sensors ranges from 150 to 400 °C in general, which can cause high power consumption [8,15]. It is also harmful to the reliability of integrated sensors. Ordered mesoporous materials may be a solution as they can improve selectivity in high-temperature environments [16,17,18]. Wang et al. [19] synthesized hierarchical Cr-doped WO_3_ microspheres and achieved a significant improvement towards H_2_S in 80 °C. Some researchers tried to use hierarchical metal oxides and binary metal oxides to solve this problem. Joshi et al. [20] prepared hierarchical NiCo_2_O_4_ structures and improved the response to O_3_ gas. Additionally, they also researched the feasibility of binary metal oxides in gas sensing. The yolk-shelled ZnCo_2_O_4_ micro–nanostructure was proved to have a fast response and shorter recovery time to 80 ppb O_3_ gas [21].

In order to demonstrate the energy dependence of the dynamical barrier and grasp the key points for MOS gas sensors, we pay great attention to the interfacial properties of gas-sensing materials and nanostructures for enhancing sensitivity, responsivity, and recovery time. Many methods have been developed to enhance the property of MOS gas sensors such as noble metal modification [22], doping [23], and core–shell (C-S) nanostructure [24]. Although these methods have been mentioned in some articles, the concepts and application are introduced briefly [4,15,18]. Differently, we summarize the gas sensing performance of various interface structures based on the sensing mechanism of material interface. Meanwhile, we have classified these methods according to the mechanisms to help readers further understand the types of gas sensors. For example, C-S nanostructure gas sensors could be summarized as heterojunction gas sensors. We summarize these gas-sensing methods and adsorption mechanisms in this review article. In addition, the main properties of MOS gas sensors based on micro–nanomaterials are discussed. Finally, some perspectives for the development of MOS gas sensors are proposed in this article.

## 2. The Properties of MOS Gas Sensors

The gas-sensing properties of MOS gas sensors are evaluated by the response [15], selectivity [17], and stability [25]. Generally, response represents the ability of gas sensors to detect target gas concentrations [26]. The resistance in air is named Ra, while the resistance exposed to the target gas is named Rg. Ia is the current in the air, and Ig is the current exposed to the target gas [27]. “a” is short for air, and “g” is short for target gas [28]. Response is expressed as the ratio of Ra and Rg, or the change in Ia and Ig [29]. Similarly, the response is described as the change in currents in the target gas to air for FET [30]. Selectivity is the ability of the gas sensors to detect one or more target gases in a mixture of gases [25,31]. Stability is the ability of a gas sensor to reproduce the results for a certain period [32]. Stability is one of the key properties of sensor devices, which is related to whether the device can effectively detect toxic gases over a long time in the detection process. Moreover, there is also recovery time, response time, and LOD (limit of detection, which expresses the smallest concentration of the target gas).

## 3. The Methods to Improve the Properties

It is essential to improve the properties on account of the extensive research for MOS gas sensors. The methods to improve the properties of materials can be divided into six aspects on the basis of our research. They consist of a change in nanostructure morphology, noble metal decorating, doping, C-S nanostructures, carbon nanomaterials, conducting polymers, 2D metal dichalcogenides, temperature modulating, heating and ultraviolet irradiation (UV irradiation).

### 3.1. Changing the Morphology of Nanostructures

This method is used to improve response or selectivity of gas sensors by changing the surface-to-volume ratios. The preparation process is usually used the hydrothermal method, CVD, ALD technique, etc. [33,34,35]. The morphology of nanostructures has been classified into four kinds: zero-dimensional nanostructures [33], one-dimensional nanostructures [36,37], two-dimensional nanostructures [38], and three-dimensional nanostructures [39]. Next, we will focus on several typical types of nanostructures.

#### 3.1.1. Nanoparticles

**Figure 1:**
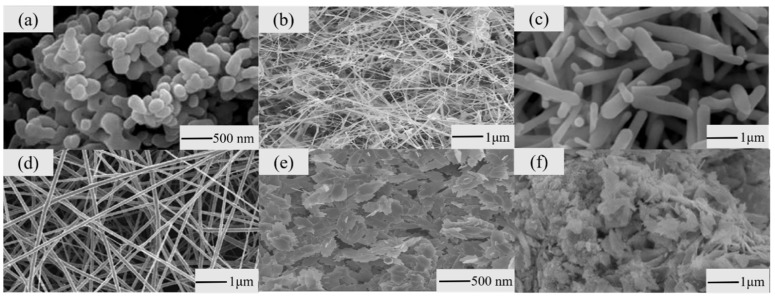
Typical nanostructures. (**a**) Nanoparticles. Adapted from [33] copyright (2015), with permission from Elsevier. (**b**) Nanowires. Adapted from [34] copyright (2008), with permission from Elsevier. (**c**) Nanorods. Adapted from [36] copyright (2014), with permission from Elsevier. (**d**) Nanofibers. Adapted from [37] copyright (2009), with permission from Elsevier. (**e**) Nanosheets. Adapted from [38] copyright (2010), with permission from Taylor & Franics. (**f**) Nanoflowers. Reprinted from [39].

Nanoparticles have higher surface-to-volume ratios, which is the cause of the deep research of nanoparticles in nanostructures. Li et al. [33] synthesized α-Fe_2_O_3_ nanoparticles via a hydrothermal reaction and calcination treatment (Figure 1a). The α-Fe_2_O_3_ nanoparticles could detect H_2_S gas whose concentration is 0.05 ppm at 300 °C.

#### 3.1.2. Nanowires

Nanowires are representative of one-dimensional nanostructured materials. Liu et al. [34] used a chemical thermal evaporation method to manufacture Ga_2_O_3_ nanowire gas sensors (Figure 1b). The experimental results indicated that the response to 5 ppm O_2_ was 10 at 300 °C, and the response to 500 ppm CO was 5 at 100 °C.

Networked nanowires are also an effective method to enhance sensing properties. Park et al. [40] succeeded in synthesizing ZnO networked nanowires using thermal oxidation of ZnSe nanowires. Single-crystal ZnO nanowire gas sensors were compared with networked nanowire gas sensors at 300 °C and 10 ppm NO_2_; the responsivity of the latter was 237, and that of the former was only 6.5. When the concentration of NO_2_ was 10 ppm, the recovery time of multinetworked ZnO nanowire gas sensors was shorter (about 180 s), and that of single-crystal ZnO nanowire gas sensors was 510 s.

Some researchers have used UV irradiation to improve the properties of nanowire gas sensors. A ZnO nanowire gas sensor was synthesized on a plastic substrate to detect ethanol gas by Lin et al. [41]. Under UV irradiation, it detected ethanol gas at 60 °C and achieved the purpose of reducing power consumption. The principle can be explained as follows: UV irradiation provided the power required for oxygen ions to reduce the operating temperature to room temperature (RT). On the one hand, the absence of nooks and crannies in nanowire-based devices contributes to the direct adsorption/desorption of gas molecules from the surface of 1D nanomaterials structures [41]. On the other hand, the bent morphology of nanowires is suitable for manufacturing flexible gas sensors. In one-dimensional nanostructures, nanowires are a research hotspot. This is due to the morphology advantages of nanowires.

#### 3.1.3. Nanorods

Nanorods are typical one-dimensional nanostructures. They usually exhibit the form of a nanorod array. In contrast to networked nanowires, nanorod arrays have a higher longitudinal orientation and better field electron emission properties [42].

**Figure 2:**
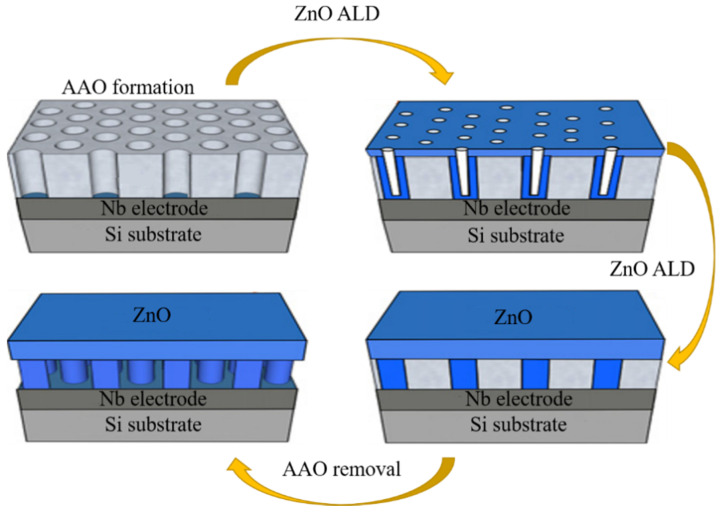
Schematic diagram of ZnO array fabricated by ALD technology. Reprinted from [35] copyright (2013), with permission from Elsevier.

Lim et al. [35] reported a vertical ZnO nanorod array on the Nb electrode by a two-step method. First, Al film and Nb films were thermally evaporated on a Si substrate. Then, they fabricated an AAO (deblock copolymers, polycarbonates, and anodic aluminum oxides) nanotemplate with several vertical pores using the chemical etching method. Finally, they used ALD techniques (atomic layer deposition techniques) to deposit ZnO film and finish the vertical ZnO nanorod array (Figure 2). It had a higher response to H2 at 350 °C. The response to 5 ppm H2 was 21 and to 500 ppm was 162 at 350 °C.

Aside from ALD techniques, Zhang et al. [36] used a ZnO nanorod array fabricated by post-annealing treatment to realize the detection of H_2_ (Figure 1c). At 425 °C, the gas sensing response was 3.56 corresponding to the H_2_ concentration of 25 × 10^−6^. They also proved that post-annealing treatment improved the crystal quality and enhanced the H_2_ gas sensing properties [36].

#### 3.1.4. Nanofibers

Nanofibers are another group of important one-dimensional nanostructures. Zheng et al. [37] used electrospinning to synthesize In_2_O_3_ nanofiber gas sensors for ethanol gas. At 300 °C and 10–500 ppm ethanol, the response was fast (1 s) and the recovery time was short (5 s). Their morphology was characterized by SEM and TEM (Figure 1d). The nanofiber structure was beneficial for ethanol molecule conduction and improved the rate at which carriers passed through the barriers [37]. Katoch et al. [43] manufactured SnO_2_ and ZnO nanofiber gas sensors by electrospinning. The response of ZnO nanofibers was higher (the response of ZnO nanofibers was 63.8, and the response of SnO_2_ nanofibers was 5.9.) in the experiment of detecting up to 10 ppm H_2_. The surface metallization of ZnO nanograins induced by H_2_ may be the reason for the enhancement of their properties.

#### 3.1.5. Nanosheets

Recently, two-dimensional nanostructures such as nanosheets have entered the view of researchers. Nanosheets can provide more adsorption sites and strong connections allowing more channels for electron transfer [44]. Hexagonal ZnO nanosheets, whose thickness was 17 nm, were synthesized by Guo et al. [45]. At 350 °C, this sensor, synthesized the using hydrothermal method, had a short response time (9 s) and recovery time (11 s). The response to up to 50 ppm formaldehyde gas was 37.8. Jia et al. [38] also used the hydrothermal method to synthesize monodisperse and stable CuO nanosheets (Figure 1e). The response to ethanol was about 3.

#### 3.1.6. Nanoflowers

Nanoflowers are layered, three-dimensional nanostructures that can effectively increase the contact area to enlarge the reaction between the target gas and sensor. This is helpful in promoting the property of gas sensors. Song et al. [39] synthesized a SnO_2_ nanoflower gas sensor to detect methanol gas via the hydrothermal method and calcination method (Figure 1f). The response of the sensor to methanol gas with a concentration of 100 ppm was about 58 at 200 °C. The response time and recovery time were 4 and 8 s, respectively, at the same temperature. The sensing mechanism can be summarized as follows: Oxygen or air seized free electrons from it and turned them into oxygen ions, and the electron depletion layer was generated with it. The electron depletion layer caused resistance to rising. When the sensor was exposed to methanol gas, oxygen ions with methanol gas reacted and released electrons into the layered SnO_2_ nanoflower. This process reduced the thickness and resistance of the electron depletion layer. The change in resistance usually expresses the responsivity of a sensor. The delamination, adsorption site, and contact area of nanostructures can greatly promote the reaction between oxygen and methanol gas. The significant change in the electron depletion layer was due to the increase in adsorption position and contact area.

In this section, we briefly introduce several nanostructures. Compared with the traditional structure of gas sensors, they can increase the absorption part of gas and the surface-to-volume ratio, so as to improve the performance of gas sensors. However, the thermal stability of special nanostructures is a significant problem. When the characteristic size is on a nanometer scale or smaller, the melting temperature of MOS will decrease [46]. In this case, nanostructures can be deformed or damaged. Moreover, zero-dimensional nanostructures have the largest ratio of surface to volume; the nanostructure stability is the worst owing to the smallest characteristic size. The feature size should be increased appropriately to prevent damage in the application process. The characteristic sizes of two-dimensional and three-dimensional nanostructures are larger than those of zero-dimensional nanostructures. Their structure stability is better than that of zero-dimensional nanostructures. When these nanostructures are close to each other, the adsorption sites could be sheltered owing to their complex morphologies [39,44,45]. Thus, we need to prevent them from forming clusters and hindering the gas adsorption in the manufacturing process.

However, the main limitation of nanoflower gas sensors is stability over their long-term operation of bending and stretching [47]. Thus, we should improve the synthetic technology of nanoflowers and add some other materials to enhance structural stability. In summary, the structural and thermal stability of nanostructures are important in the fabrication of gas sensors. In addition, the nanoflowers’ structure may have the most potential for the application of gas sensors due to the huge surface area.

### 3.2. Noble Metal Decorating

This method mainly depends on the electron sensitization and chemical catalysis of noble metals on the interface of materials [48,49]. Noble metal decorating can validly enhance the responsivity and selectivity of MOS sensors [50,51,52,53,54]. Some noble metal particles also increase recovery time [55,56,57]. When the surface of the material is decorated with a noble metal, some chemical reactions often occur at the micro level, while the change of resistance structure is observed at the macro level [58,59,60]. According to these changes, we often classify sensors based on this method as chemical resistance sensors [60,61]. We demonstrate some types of noble metal nanoparticles and some target gases in Table 1. In the next sections, we use the example of NO_2_ gas to describe the effect of noble metal nanoparticles [56,59,60].

Decorating (loading) with Au and Ag nanoparticles could improve response and selectivity. For example, Liang et al. [56] and Zhang et al. [62] used Au nanoparticles to decorate a VO_2_ nanowire sensor and bilayer WO_3_ nanoporous thin-film sensor, respectively. This was useful to improve response and selectivity for NO_2_ gas. Moreover, decoration with Ag nanoparticles had the same effect. Kamble et al. [59] improved the performance of WO_3_ film in sensors by modification with silver nanoparticles. The response speed was increased by 6 times. Xiao et al. [26] manufactured a Ag-In_2_O_3_ nanosphere sensor. The best response was 58 toward NO_2_ gas with a concentration of 10 ppb, while the pristine In_2_O_3_ nanospheres’ response was 25.5 at 120 °C. The selectivity of the sensor to NO_2_ was more significant than that to some volatile organic compounds (VOCs). Additionally, this sensor exhibited several responses under different concentrations of NO_2_ and proved that decoration with Ag nanoparticles can reduce recovery time.

**Figure 3:**
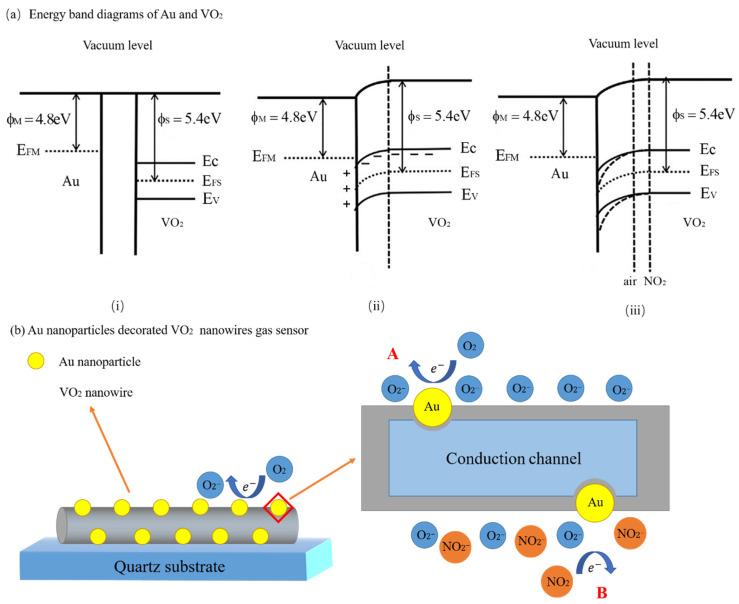
Schematic diagram of the mechanism of Au nanoparticles [56]. Reprinted from [56] copyright (2018), with permission from Elsevier. (**a**) The energy band diagrams of Au and VO_2_; (**b**) The gas sensing mechanism of Au nanoparticles.

The performance of MOS gas sensors can be improved by modification with metal materials through electron sensitization and chemical sensitization. The electronic sensitization mechanism improves the response of MOS gas sensors in two ways. On the one hand, when noble metal nanoparticles contact MOS nanomaterial, their Fermi levels will be aligned together [58]. Due to the different work functions, electrons flow in their energy bands, causing their energy bends to be bent. When the Fermi level arrives at a new balance, the depletion region and the Schottky barrier will be created in the interfaces (Figure 3) [56]. They influence the concentration of carriers or improve the mobility of carriers [58,59]. In these circumstances, the baseline resistance changes, and the sensor response is improved. On the other hand, when noble metal nanoparticles were decorated on the surface of the gas sensor nanomaterial, adsorption sites were increased, and the rate of gas adsorption was accelerated. In contrast to the electronic sensitization mechanism, the chemical sensitization mechanism can be defined as the catalysis of noble metals [63]. The activation energy of the reaction between iron oxide and the target gas can be reduced by using gold nanoparticles as a catalyst [64]. The chemical sensitization mechanism is also known as the spillover effect [64]. Furthermore, Pt and Pd nanoparticles can be used to decorate the surface of a MOS to improve the properties. More details can be found in [53] and [65]. To reduce costs, we can decorate with transition metal oxide nanoparticles instead of the noble metal nanoparticles to enhance the properties. Na et al. [66] decorated ZnO nanowires with Co_3_O_4_ nanoparticles. This improved response and selectivity to NO_2_ and C_2_H_5_OH. Ko et al. [67] synthesized SnO_2_ nanowires decorated with V_2_O_5_ nanoparticles with a better response to NO_2_. The mechanism of the transition metal oxide nanoparticles is characterized by the form of heterojunctions. With the help of heterojunctions, the electron depletion layer and the mobility of carriers obtain modulation. Then, baseline resistance can be altered. Based on this, response and selectivity were improved.

In summary, improving the performance of the gas sensors via the electronic sensitization and chemical sensitization of the noble metal is very effective. The sensitization mechanism with the catalytic effects has been pointed out in our article. Furthermore, the impact on the selectivity of gas sensors depends on the type of noble metal. For instance, Au nanoparticles show good selectivity for NO_2_ or CO [23,31], while Ag nanoparticles are sensitive to NO_2_ or ethanol [26,58]. A noble metal with sensitive materials has the possibility of forming a cluster and hindering gas adsorption. Therefore, we should uniformly disperse the noble metal nanoparticles on the surface of host-sensitive materials [67,68,69]. Considering the structural stability, the noble metal is more easily destroyed than the host-sensitive nanostructure owing to the size of nanoparticles. Thus, we should pay attention to the preparation process of the noble metal decorating, the operation temperature, and the thermal stability of gas sensors [49,53,56,60].

### 3.3. Doping

In addition to noble metal decorating, doping can also be used to improve the properties of chemical resistance gas sensors. Some researchers have used doping to increase MOS sensor properties, such as response, response time, and recovery time [27,70]. Metal oxide [27], metal [28,71], nonmetallic elements [70,72], and so on can be used as dopants.

Han et al. [27] reported a self-doped nanocolumnar vanadium oxide gas sensor. Due to the effect of self-doping, response and selectivity were both enhanced to NO_2_ gas. Bayata et al. [28] synthesized an Al-doped titania gas sensor. The best response to hydrogen was acquired under 300 °C. The response time and recovery time were shortened to different degrees. Moreover, more adsorption sites occur due to doping. Yu et al. [71] produced a 2% Al-doped ZnO nanovase gas sensor. Compared to the pristine ZnO nanovase gas sensor, the produced sensor had higher response and selectivity to CO, and its response time was shortened. Basu et al. [70] fabricated an F-doped SnO_2_ film gas sensor. Its response time and recovery time were shortened to 22 and 52 s, respectively. The mechanism can be described in that dopants can modulate the concentration of carriers or expand the width of the electron depletion layer to change baseline resistance or conductivity.

Compared with noble metal decorating, dopants cannot form a cluster on the surface of host-sensitive nanostructures. Moreover, dopants can not only decrease the activation energy and control the specific exposed facets but also lead to a catalysis effect [69]. However, excessive doping may cause poor electron mobility [72]. Therefore, we need to monitor the number of dopants to avoid the adverse influence of excessive doping.

### 3.4. Core-Shell (C-S) Nanostructures

#### 3.4.1. Overview

##### The Definition of C-S Nanostructure

C-S nanostructure is a special nanocomposite and plays an important role in gas sensing [31]. It is usually composed of a core nanomaterial and a shell nanomaterial covering the core. Compared with non-C-S structures, C-S nanostructure provides a way to maximize the interfacial area between two or more materials [73]. In addition, C-S nanostructures can protect the core nanomaterial from the surrounding environment, so as to improve physical and chemical properties [74].

**Figure 4:**
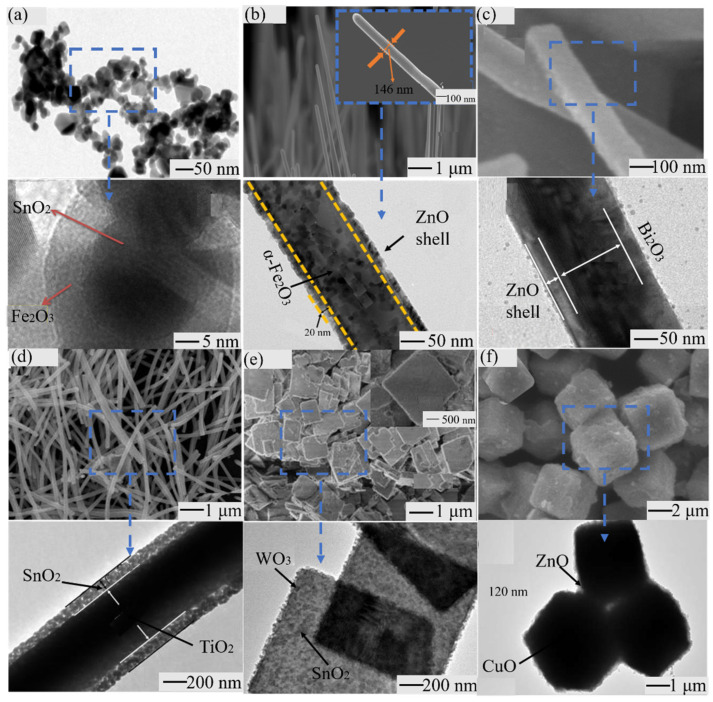
Typical C-S nanostructures. (**a**) C-S nanoparticles. Adapted from [75] copyright (2019), with permission from Elsevier. (**b**) C-S nanowires. Adapted from [76] copyright (2020), with permission from Elsevier. (**c**) C-S nanorods. Adapted from [81] copyright (2017), with permission from Elsevier. (**d**) C-S nanofibers. Adapted from [82] copyright (2017), with permission from Elsevier. (**e**) C-S nanosheets. Adapted from [86] copyright (2018), with permission from Elsevier. (**f**) C-S microcubes. Adapted from [87] copyright (2016), with permission from Elsevier.

Due to above, C-S nanostructure has been applied in zero-dimensional nanomaterials (nanoparticles [75]), one-dimensional nanomaterials (nanowires [56,76,77,78], nanorods [79,80,81], nanofibers [82,83,84,85]), two-dimensional nanomaterials (nanosheets [86]), and three-dimensional nanomaterials (microcubes [87]). Figure 4 shows these C-S nanostructures. Because different C-S nanostructures have diverse applications, their material combinations are also different.

##### The Composition of C-S Nanostructures

The composition of C-S nanostructure materials can be roughly divided into the following categories: noble metal/noble metal [85,88], metal oxide/metal oxide [31,89,90,91], metal oxide/metal sulfide [92,93], and metal oxide/noble metal (including core shell exchange) [94,95]. Next, we introduce some typical examples.

(1) Metal oxide/metal oxide

Metal oxides are usually applied in metal oxide semiconductors (MOSs) as important functional materials in gas sensing. MOSs are typically divided into n-type MOSs with the electron as the carrier and p-type MOSs with the hole as the carrier. When oxidizing gas acquires electrons contacting an n-type MOS, the concentration of electrons in the n-type MOS will decrease and conductivity will weaken. When reducing gas releases electrons contacting an n-type MOS, the concentration of electrons in the n-type MOS will increase and conductivity will be enhanced. However, the conductivity of p-type MOS is contrary to that of the n-type MOSs, as shown in Table 2. MOS conductivity will have different changes in different gas environments. Thus, we can choose relevant a MOS to form the heterojunction at the interface of C-S nanostructures such as p–n heterojunction [96,97], n–n heterojunction [80], or p–p heterojunction [98]. The mechanism and cases will be demonstrated in the section on applications of the C-S nanostructure.

(2) Metal oxide/metal sulfide

The metal oxide/metal sulfide combinations are similar to the metal oxide semiconductor material combinations. Different types of semiconductor (n-type, p-type) materials are used to form the corresponding heterostructure (such as p–p heterojunction) to improve the properties of the sensor. The mechanism and cases are demonstrated in the section on applications of the C-S nanostructure.

(3) Metal oxide/noble metal

The catalytic effect and the carrier mobility are amplified due to the participation of noble metal nanoparticles. The mechanism and cases are demonstrated in the section on applications of the C-S nanostructure.

#### 3.4.2. The Thickness of Shell Layer

In the C-S nanostructure, the types of MOS (p-type and n-type) that constitute the core and shell layer can be chosen according to the type of target gas (oxidizing gas or reducing gas). However, the thickness of the shell layer will affect the response of the sensor.

**Figure 5:**
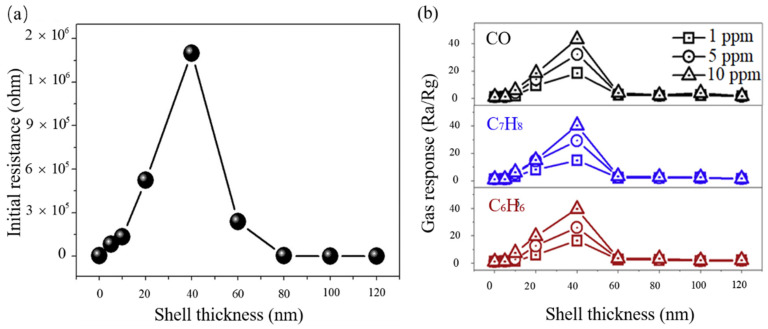
The influence of shell thickness of sensor response. Reprinted from [77] copyright (2020), with permission from Elsevier. (**a**) Relationship between the Initial resistance and the shell thickness; (**b**) The dynamic curve of gas response with shell thickness.

Kim et al. [77] investigated the effect of the thickness of the shell layer on sensor response under different gas concentrations. First, they measured the dynamic resistance changes of ZnO-SnO_2_ C-S nanowire gas sensors with different thicknesses of the SnO_2_ shell layer. The dynamic curve of resistance changes showed that the sensor has the properties of an n-type semiconductor for detecting the C_6_H_6_, C_7_H_8_, and CO gas with concentrations of 1, 5, and 10 ppm respectively. Then, they explored how the thickness of the SnO_2_ shell layer affects the ZnO-SnO_2_ C-S nanowire gas sensor’s properties. The shell thickness of 40 nm is an important parameter for the gas sensing performance of ZnO-SnO_2_ materials, which can be seen in the bell curve in Figure 5. In short, though the mechanisms of all kinds of C-S structures are different, there is an optimum shell thickness where the response will arrive at the peak [77]. The influencing factor of the optimum shell thickness is the Debye length (λD) of the shell layer. When the shell layer’s thickness is close to the Debye length (λD) of the shell layer, an ideal response will be acquired. Besides, many articles relate the shell layer’s thickness with the Debye length [81,99,100].

The Debye length, also known as the Debye radius, is a typical length describing the action scale of charge in plasma and is an important parameter of plasma. The mechanism of a C-S nanostructure is relevant to the changes in the electron depletion layer. The change of the electron depletion layer is also related to the Debye length. Taking reducing gas as an example, when reducing gas reacts with adsorbed oxygen ions and the shell material, several electrons will be released to the electron depletion layer. If the shell layer’s thickness is smaller or equal to the Debye length, the electron depletion layer can be changed from the whole electron depletion layer to the part electron depletion layer. It will cause a significant change in resistance, allowing a high response to be acquired [77]. If the shell layer thickness is greater than the Debye length, the initial electron depletion layer will not be wholly depleted. When released electrons contact the shell material, the resistance change will not be evident, and the response will not be higher [99]. In short, when the shell layer’s thickness is close to the Debye length, a significant response can be acquired.

#### 3.4.3. The Manufacture of C-S Nanostructures

ALD techniques [99,101] and coaxial electrospinning [102,103,104,105] are used to produce C-S nanostructures. Atomic layer deposition techniques are abbreviated as ALD techniques. The process is usually composed of several cycles. Every cycle includes precursor pulse, reactant pulse, and purification [31]. Users can change the number of cycles to control the shell layer’s thickness. There are numerous advantages to ALD techniques, especially the precise control of the thickness and the uniform coverage ability [101]. Coaxial electrospinning is another method to produce C-S nanostructure gas sensors. The coaxial electrospinning device is mainly composed of a high-voltage DC power supply, liquid supply system, composite nozzle, and collecting plate [103].

The process is as follows: First, the required solution is mixed, and magnetic stirring is used to finish the manufacture of the precursor solution. After that, the precursor solution is put into the electrospinning syringe and then ejected through the composite nozzle. At last, the finished C-S nanostructure is collected [102,104]. The main advantages of coaxial electrospinning are simple synthesis and reliable structure [105]. Besides, there are numerous methods such as the hydrothermal method [106], coprecipitation, and sol–gel processes that can be used to prepare a core–shell nanostructure [75,78].

#### 3.4.4. The Application of C-S Nanostructures

C-S nanostructures have been researched to detect inorganic gas and VOCs (Table 3). To further introduce the application of C-S nanostructures, we will summarize the research progress of C-S nanostructure gas sensors of three types: metal oxide/metal oxide [31,89], metal oxide/metal sulfide [92,93], and metal oxide/noble metal [94,107].

##### Metal Oxide/Metal Oxide

The p–n heterostructures, which are composed of n-type and p-type MOSs, are widely researched C-S nanostructures in gas sensing.

Liang et al. [108] produced ZnO-NiCo_2_O_4_ C-S nanofibers via a chemical deposition method. Their response time and recovery time towards methanol were 37 and 175 s, respectively. Those of pristine ZnO nanofibers were 123 and 338 s. The responses to methanol of 5, 10, 20, 50, and 100 ppm were 1.96, 3.02, 3.97, 4.88, and 6.77, respectively, which were much higher than those of pristine ZnO at the same concentrations. The improvement of the property was due to the p–n heterojunction and the unique C-S porous structures. Li et al. [89] synthesized a ZnO-Co_3_O_4_ C-S nanostructure. At the best temperature (200 °C), the response to ethanol (100 ppm) gas was 38.87. Compared with single shell ZnO-Co_3_O_4_ nanostructure, the best temperature had decreased 40 °C and the response had increased 25.07. Majhi et al. [109] produced a PdO-ZnO C-S nanostructure gas sensor to detect acetaldehyde gas. Due to p–n heterostructure, at 350 °C, the best response was 76 to acetaldehyde gas with 100 ppm, while that of the pristine ZnO nanostructure was 18. Moreover, PdO nanoparticle catalytic behavior was not ignored. That is why the response time was shortened to 20 s. Xu et al. [110] anchored NiO porous nanosheets on α-MoO_3_ nanobelts to synthesize α-MoO_3_-NiO-2 C-S nanobelts and α-MoO_3_-NiO-1 C-S nanobelts. The α-MoO3-NiO-2 and α-MoO_3_-NiO-1 are differentiated by the content of anchored NiO. Their responses to acetone were 17.2 times and 16 times greater than those of the pristine α-MoO_3_ structure. Compared with pristine NiO structure, α-MoO_3_-NiO-1 C-S nanobelts’ response was 6.6 times higher. According to their experiment, though pristine NiO nanosheets had much higher surface-to-volume ratios, the improvement of properties depended on the heterostructure. Kim et al. [31] also studied the effect of p–n heterostructure on NO_2_. They used ALD techniques to produce SnO_2_-Cu_2_O C-S nanofibers. When the thickness of Cu_2_O was 30 nm and the concentration of NO_2_ was 10 ppm, SnO_2_-Cu_2_O C-S nanofibers’ response time and recovery time had been shortened by 137 and 46 s compared to the pristine SnO_2_ nanofibers.

Besides, n–n heterostructure and p–p heterostructure have also been produced with C-S nanostructures. Jayababu et al. [111] synthesized CeO_2_-Fe_2_O_3_ C-S nanoparticles via the sol–gel method. Their response time and recovery time towards 100 ppm of ethanol at RT were 3 and 7 s. Compared with pristine CeO_2_ nanoparticles and pristine Fe_2_O_3_ nanoparticles, the C-S nanostructure’s properties were enhanced. The main cause of enhanced properties is the n–n heterostructure. The catalytic behavior of Fe_2_O_3_ nanoparticles also helped in the shortening of response/recovery time. In Yin et al.’s work [106], a WO_3_-SnO_2_ nanosheet was synthesized by the hydrothermal method, and its properties and sensing mechanism were investigated. Taking the WO_3_-SnO_2_ nanosheet whose particle density of SnO_2_ was 0.5% as an example, the working temperature decreased by 80 °C and response time shortened by 3 s. On the one hand, the n–n heterostructure and the modulation of barrier height improve properties. On the other hand, the interface between WO_3_ nanosheets and SnO_2_ nanoparticles improved, which accelerates the reaction of target gas and sensor [106]. Wan et al. [112] used the hydrothermal method and electrospinning to produce In_2_O_3_-SnO_2_ C-S nanofibers. At 120 °C, their response to formaldehyde gas with a concentration of 100 ppm reached 180.1. Compared with pristine In_2_O_3_ nanofibers and SnO_2_ nanofibers, this represents an increase by 9 times and 5 times, respectively. Diao et al. [90] synthesized ZnO-CeO_2_ nanofibers to detect acetone gas. The best operation temperature was 370 °C. At this temperature, the response arriving at the peak was 8.2. Moreover, the nanostructure can detect target gas at a lower concentration. At 0.2 ppm acetone, the response was 3.8. In Wang et al.’s work [98], p–p heterostructure was introduced to detect H_2_S. The response of the CuO-NiO C-S microspheres designed by them was 47.6 at 260 °C. It almost was 3 times greater than that of pristine CuO microspheres. The mechanism can be summarized as follows: p–p heterostructure, wrinkles on the surface of the NiO shell layer, and the catalysis of the NiO shell layer.

The C-S nanostructure is a typical heterostructure. The mechanism of this kind of C-S nanostructure gas sensor is mainly attributed to the formation of the electron depletion layer or the hole depletion layer and the modulation of barrier height [101,106,111]. When two nanomaterials contact each other, the heterostructure will form at the interface between them [113]. When p-type MOS and n-type MOS contact with each other, p–n heterostructure, p–p heterostructure, or n–n heterostructure will be formed. Usually, the compositions of the core layer and shell layer are different, so the work functions are different. The work function is determined by the composition of the Fermi level and the electron depletion layer or the hole depletion layer at the interface of the heterostructure [113]. To balance the Fermi Level, when MOS materials contact each other, charges will transfer at their interior. With the transfer of charges, the barrier height will change, and the electron depletion layer or the hole depletion layer will arise on the side of the output electrons.

**Figure 6:**
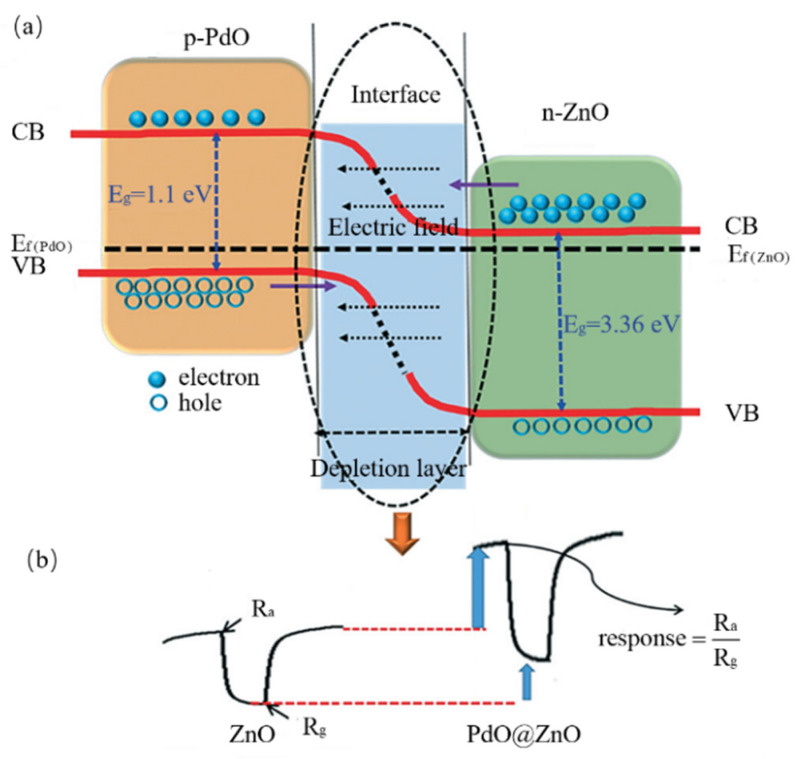
Schematic diagram of the mechanism of core-shell structure. Reprinted from [109]. (**a**) The energy band diagrams of PdO-ZnO heterojunction; (**b**) Response signals of the prisitine ZnO nanostructure and PdO-ZnO heterojunction.

Generally, the process by which the electron depletion layer or the hole depletion layer and the barrier height affect the response of the sensor is divided into three parts. Taking the PdO-ZnO C-S nanostructure gas sensor made by Majhi et al. [109] as an example, we will demonstrate this process. First, the work function of PdO (7.9 eV) is higher than the work function of ZnO (5.3 eV). As an effect, the electrons in the conduction band of ZnO move to the conduction band of PdO, and the hole moves to ZnO. When the Fermi level of this system is balanced, an electron depletion layer will be formed near ZnO, and a hole depletion layer will be formed near PdO at the interface of the PdO-ZnO heterojunction. This also can lead to band bending (Figure 6). Then, if this sensor is exposed to oxygen or air, the oxygen will be absorbed by the surface of the sensor, and oxygen molecules will change into oxygen atoms. Some of the oxygen atoms will trap electrons from the conduction band of ZnO and form oxygen ions. This process is described by Equations (1) and (2).

O_2_(gas) → 2O(ads), (1)

O(ads) + e^−^(from ZnO) → O^−^(ads), (2)

This process will produce a new electron depletion layer at the surface of ZnO nanomaterials. With the effect of oxygen ions, the width of the electron depletion layer at the PdO-ZnO heterojunction could be increased. As the electron depletion layer changes, high potential barriers hinder electron transfer and produce high resistance between ZnO and PdO nanoparticles. In this process, Ra denotes the pristine resistance of this sensor. At last, when the sensor is exposed to acetaldehyde gas, those absorbed oxygen ions will react with acetaldehyde gas and release trapped electrons to the conduction bands of PdO and ZnO. The barriers between ZnO nanoparticles and PdO nanoparticles will have decreased, and the width of electron depletion layers will also have decreased. Owing to the change of electron depletion layers and the modulation of barrier height, resistance will have been reduced. Rg describes the ultimate resistance of this sensor. We can obtain the final response by the radio of Ra and Rg. In this kind of C-S nanostructure gas sensor mechanism, the depletion layers and barrier height have a variety of different changes in oxygen or air. Table 4 summarizes the change of depletion layer thickness for oxidizing gas or reducing gas.

##### Metal Oxide/Metal Sulfide

ZnO is one of the popular materials in gas sensing. Pristine ZnO displays n-type properties because of oxygen vacancies and Zn interstitial atoms. However, researchers doping other elements such as nitrogen change n-type ZnO to p-type ZnO. Aside from metal oxide, parts of metal sulfide can be applied to gas sensing.

Chang et al. [92] used p-type ZnO and MoS_2_ to produce a p-ZnO-MoS_2_ C-S nanosheet gas sensor by the hydrothermal method. Firstly, they tested this sensor’s response to 500 ppb acetone gas at 350 °C. It was 18 times that of pristine p-ZnO. Secondly, in a low concentration acetone experiment (100 ppb), it had a near 80 times increase in response compared to pristine p-ZnO and pristine MoS_2_. Additionally, the recovery time and response time were shortened. They succeeded in detecting ultra-low concentration acetone gas. This sensor not only detected 5 ppb acetone but also possessed a fast response time and recovery time (60 and 40 s). Its mechanism is based on the change of depletion layers. When exposed to air, oxygen will trap electrons from the surface of p-type ZnO. In this process, the hole concentration will increase, and the hole depletion layer will be reduced at the interface of p-ZnO-MoS_2_ heterojunction. This decreases the pristine resistance (Ra). If the acetone gas contacts it, oxygen ions will react with acetone gas and release electrons to the conduction band. With the generation of electrons, the hole concentration will decrease, and the hole depletion layer will expand. The sensor’s ultimate resistance (Rg) will increase. According to Chang et al. [92], the response is described by Equation (3).

Response = (Rg − Ra)/Ra × 100%(3)

In their latest article, UV irradiation was introduced to hollow p-ZnO-MoS_2_ C-S nanosheets [93]. Compared with hollow p-ZnO-MoS_2_ C-S nanosheets, UV-irradiated hollow p-ZnO-MoS_2_ C-S nanosheets’ response to 20 ppm acetone had increased 2.32 times at 100 °C. The UV-irradiation mechanism can be summarized as follows: it can induce more electron–hole pairs, which is helpful to oxygen adsorption and the rate of oxygen reacting with electrons.

##### Metal Oxide/Noble Metal

In addition to the combination of the two types mentioned earlier, the metal oxide–noble material combination is also promising for core-shell nanostructures.

Majhi et al. [94] synthesized Au-NiO C-S nanoparticles by wet chemical methods at 85 °C. Compared with pristine NiO nanoparticles, operation temperature dropped from 300 to 200 °C, the response to 100 ppm ethanol increased from 1.68 to 2.54 at 200 °C, the response time decreased from 400 to 250 s, and the recovery time decreased from 540 to 420 s. The sensing mechanism can be attributed to the formation of the Schottky junction and the catalysis of metal particles. Yang et al. [78] prepared a kind of Ag-TiO_2_ C-S nanowires. The response of these nanowires to ammonia was 200 at 240 °C. Compared with pristine TiO_2_ nanowires, the operation temperature decreased by 20 °C. Besides, the response time of pristine TiO_2_ nanowires in ammonia with concentrations of 20, 50, 100, 300, and 500 ppm was 29, 30, 31, 33, and 35 s, respectively. Due to the catalysis of Au particles and the formation of the Schottky barrier, the response of this structure can be effectively shortened to 26, 28, 27, 28, and 30 s, respectively. Zhao et al. [95] prepared a NO_2_ sensor based on Au-WO_3_ C-S nanospheres. The response of the nanosphere to 5 ppm NO_2_ was 136 at 100 °C, which is 5 times the response of pristine WO_3_ nanospheres under the same conditions. The response time of the structure is 4 s, while that of the pristine WO_3_ nanosphere is 218 s, and their recovery times are 59 and 2649 s. Moreover, they verified the sensor could maintain good NO_2_ sensing performance at high humidity of 75% RH.

This combination is different from the previous methods, and the sensing mechanism is mainly attributed to the Schottky junction and the catalytic behavior of metal particles. We take the Ag-TiO_2_ C-S nanowire gas sensor designed by Yang et al. [78] as an example to summarize. First of all, it will create the Schottky junction on the contact interface because of the existence of Ag nanowires when Ag nanowires contact the TiO_2_ shell layer. Because the work function (4.3 eV) of TiO_2_ is smaller than that of Ag (4.6 eV), free electrons begin to flow. The electron depletion layer (side of the TiO_2_ shell) is generated on the contact interface between Ag nanowires and the TiO_2_ shell layer. Then, when the sensor is exposed to oxygen, the ionized oxygen ions capture electrons from the TiO_2_ layer and form adsorbed oxygen. During the process, the Ag nanowires continuously provide electrons to the surface of TiO_2_. The electron depletion layer continues to widen, and the Schottky barrier height increases, leading to an improvement in the resistance of the sensor in air (Ra). When the sensor is in contact with the reducing gas NH_3_, the ionized oxygen ions react with NH_3_. The trapped electrons are released from the surface of TiO_2_ to the Ag nanowires. Therefore, the widened electron depletion layer is gradually reduced, and the Schottky barrier height decreases. Finally, the resistance of the sensor in ammonia (Rg) decreases. The response is described by Equation (1). Besides the Schottky junction, the chemical catalytic behavior of Ag nanoparticles is also an important factor in enhancing response. Ag nanoparticles can reduce the reaction barrier between the target gas and oxygen ions and promote the surface reaction. The chemical catalytic behavior of noble metals has been discussed in detail in Section 3.2.

#### 3.4.5. C-S Nanostructure and Noble Metal Decorating/Doping

Ju et al. [114] synthesized Au-Loaded ZnO-SnO_2_ C-S nanorods by pulsed laser deposition (PLD) and DC sputtering. The response to 50 ppm of triethylamine (TEA) is about 12.4 and the response time is 1.2 s at 40 °C. The performance of core-shell nanorods is much better than that of pristine ZnO nanorods. The improvement of properties mainly depends on the Schottky junction between the Au nanoparticles and SnO_2_ shell and the n–n heterojunction between the ZnO layer and SnO_2_ layer. Kim et al. [1] loaded Au nanoparticles on the SnO_2_-ZnO C-S nanowires. When the working voltage was 5 V, the response to 0.1, 1, 10, and 50 ppm CO was about 1, 1, 1.16, and 1.25. When the working voltage was 20 V, the response reached about 1, 1.25, 1.40, and 1.62. Besides, they also verified that the formation of the Schottky barrier and the catalysis of Au nanoparticles can enhance the response to CO. Gong et al. [115] used heterogeneous precipitation and sintering treatment to prepare Ga-doped (1 mol%) Pt-ZnO C-S nanoparticles. Compared with the Pt-ZnO C-S nanoparticles without Ga doping, the Ga-doped C-S nanostructure achieved the ability to detect acetone at 10 ppb and 20 ppb. The response of this structure to 1 ppm acetone increased from 2.4 to 13.8, and the optimum operating temperature dropped to 275 °C from 300 °C. Bonyani et al. [81] decorated Bi_2_O_3_-ZnO C-S nanorods with Pd nanoparticles. The special structure of the sensor effectively shortened the response and recovery times. The response to 200 ppm benzene was 28 at 300 °C, which is higher than that for the pristine Bi_2_O_3_ (1.7) and pristine ZnO (6.8) nanorods. It was found that decorating (loading) or doping the C-S nanostructure with noble metal can improve the performance of the sensor effectively. Kim et al. [116] decorated SnO_2_-ZnO C-S nanowires with CuO; combined with the self-heating effect, this could also improve the selectivity to H_2_S.

The mechanism of this combination is mainly attributed to the synergetic effect including the Schottky junction, heterojunction, and the catalytic behavior of metal and some metal oxide particles. We take the Au-loaded ZnO-SnO_2_ C-S nanorod gas sensor to detect triethylamine (TEA) in the experimental work of Ref. [114].

**Figure 7:**
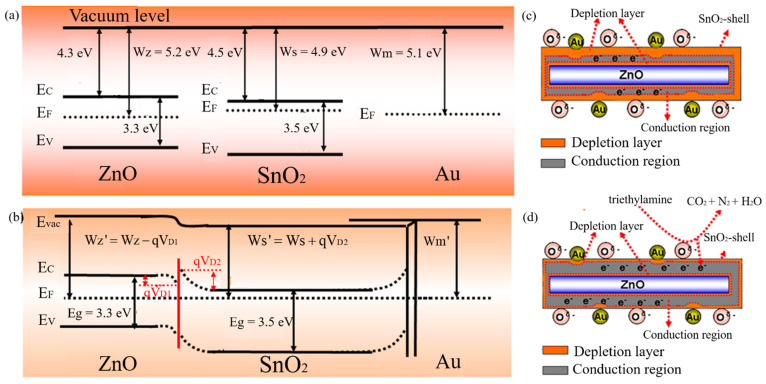
Schematic diagram of the mechanism of interaction between core–shell structure and noble metal nanoparticles. Reprinted from [114] copyright (2015), with permission from American Chemical Society. (**a**) Schematic diagram of energy bands for ZnO, SnO_2_, and Au; (**b**) Schematic diagram of energy bands for Au-SnO_2_/ZnO heterojunction; (**c**) Schematic diagram of Au-SnO_2_/ZnO sensor exposed to air; (**d**) Schematic diagram of Au-SnO_2_/ZnO sensor exposed to TEA.

Due to the influence of different work functions, the electron depletion layer is formed on the contact interface of ZnO and SnO_2_ (side of the SnO_2_ shell) when Au nanoparticles are loaded on the surface of SnO_2_-ZnO C-S nanorods. The electrons will flow from the SnO_2_ shell to Au nanoparticles, which leads to the Schottky junctions forming on the contact interface of Au and SnO_2_. Then, the flow of electrons widens the depletion layer on the SnO_2_. Besides, due to the existence of Schottky junctions and heterojunction, the electron depletion layer of the SnO_2_ shell further expands and leads to resistance improvement when the sensor is exposed to oxygen. By contrast, the trapped electrons are released from the reaction of TEA and O ions when the sensor is exposed to TEA. The electron depletion layer of the SnO_2_ shell layer is reduced, resulting in a decrease in resistance. The sensor response is described by Equation (2), where Ra and Rg are the resistances of the sensors in air and target gas, respectively. Its mechanism is shown in Figure 7. To sum up, the C-S nanostructure can effectively improve the properties of MOS gas sensors.

To sum up, the C-S nanostructure is composed of the host-sensitive nanomaterials (core layer nanostructure) and the external-sensitive nanomaterials (shell layer nanostructure). Generally speaking, the thickness of the external-sensitive layer affects C-S nanostructure properties, owing to the Debye length. Because the sensing mechanism of C-S nanostructure usually involves the heterojunction, we can roughly classify C-S nanostructure gas sensors according to the different materials of the heterojunction, namely p–n heterojunction, n–n heterojunction, and p–p heterojunction gas sensors. For MOS/MOS and MOS/metal sulfide C-S nanostructure sensors, the sensing mechanism can be summarized as the formation of heterojunction and the modulation of barrier height. As for MOS/noble metal C-S nanostructure, the mechanism also includes the catalytic behavior of metal particles. More details about the catalytic behavior of metal particles can be found in Section 3.2. Obvious advantages of C-S nanostructure gas sensor can be summarized as follows: (1) higher response due to the synergistic effect of multiple sensing mechanisms [78,79,101,106]; (2) reducing the interference of unnecessary other gases [117]; (3) protecting the host-sensitive nanostructure [87]; (4) making full use of MOS advantages. For example, the α-MoO_3_-NiO-2 C-S nanobelt gas sensor manufactured by Xu et al. displayed a high response to acetone gas and good thermal stability [107]. This is due to the p-type MOS having better thermal stability and the n-type MOS having better carrier mobility. Moreover, the critical points to consider in achieving better properties in C-S nanostructures are mainly as follows: (1) the modulation of energy barrier; (2) the catalytic behavior of other materials; (3) the mechanism of carrier mobility [68]. However, the synthesis process for C-S nanostructures is more tedious and the cost is more expensive [82]. Therefore, we should research new preparation processes to solve these problems. Besides, the external additives influence the adsorption capacity and chemical reactivity of the host-sensitive material’s surface [118], which has often been disregarded in gas sensor studies. So, when we select suitable materials to make the C-S nanostructure, we should pay attention to the interaction of the C-S interface in the context of density functional theory (DFT), molecular dynamics, or other theories.

### 3.5. Carbon Nanomaterials

In recent years, carbon nanomaterials have been widely used in the field of gas sensing due to their excellent conductivity and mechanical and thermal properties. Among the most representative are graphene and its derivatives, which have a high surface-to-volume ratio and active functional groups on the surface [119,120,121], and carbon nanotubes (CNTs) and their products with excellent electrical properties and high flexibility [122,123,124].

**Figure 8:**
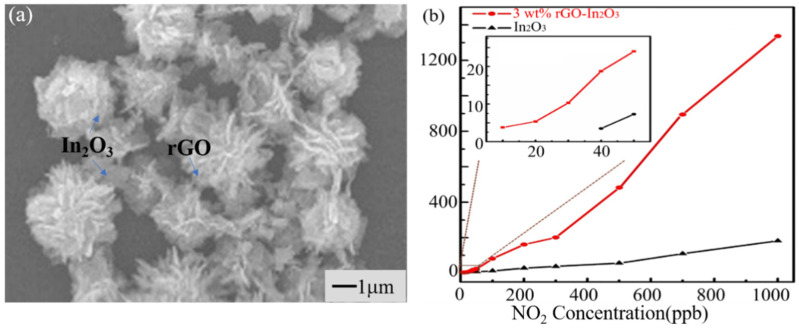
rGO-In_2_O_3_ gas sensor. Adapted from [130] copyright (2017), with permission from Elsevier. (**a**) The morphology of rGO-In_2_O_3_ composite nanostructure; (**b**) the response of rGO-In_2_O_3_ composite nanostructure to NO_2_.

There is often a limitation due to poor selectivity and high operation temperature when using conventional metal oxide semiconductor sensors. Due to its unique properties, graphene can effectively improve the selectivity and carrier mobility of the composites with the synergy of metal oxide. Wang et al. [125] synthesized a kind of nanocomposite with ZnO nanosheets and graphene oxide, effectively increasing the contact area of the target gas as it has a high surface-to-volume ratio and more gas molecular adsorption sites. Moreover, the response–recovery ability was improved compared with pristine ZnO nanosheets by modulating the barrier at the materials’ interface. Feng et al. [126] used electrospinning technology to prepare a kind of rGO-encapsulated Co_3_O_4_ composite nanofiber that can monitor ammonia at room temperature. The response of the composite nanofibers to ammonia gas was significantly higher than that of the composite without rGO, and the response to 50 ppm ammonia was above that of the other interfering gases by 10 times. The improvement of the selectivity of the nanofibers is probably attributed to two reasons: On the one hand, these polarized bonds of rGO and Co^3+^ centers had an even stronger interaction with ammonia that has one lone pair of electrons. On the other hand, the capacity of the pore walls to adsorb different gases is different when gas diffuses in the mesopores of carbon nanofibers. As a derivative of graphene, the defects in the preparation process and electrical properties of the residual oxygen components contributing to the main carrier in rGO are holes. Generally, rGO under ambient conditions exhibits p-type behavior due to the electron-withdrawing nature of defects [127]. Li et al. [128] synthesized rGO-decorated TiO_2_ microspheres by the hydrothermal method, and the p–n heterojunction formed between n-type TiO_2_ and p-type rGO enhanced the selectivity to ammonia. The heterojunction also suppressed the response to other alcohol gases. They also measured the effect of humidity on the sensor performance in the experiment: the capacity of rGO to adsorb H_2_O molecules was stronger than its capacity to adsorb ammonia molecules. With the increase in relative humidity, more water molecules covering the rGO membrane providing electrons led to the decrease in the ammonia recognition ability. The content of carbon nanomaterials in the composite materials had a significant influence on the overall sensing performance [129]. The operation temperature of NO_2_ gas was tested by adjusting the content of rGO in the composite [130]. In the composite material, the active adsorption sites increased with the increase in rGO content, which led to the increased response performance of their sensor (Figure 8). Then, the operation temperature gradually dropped to room temperature.

**Figure 9:**
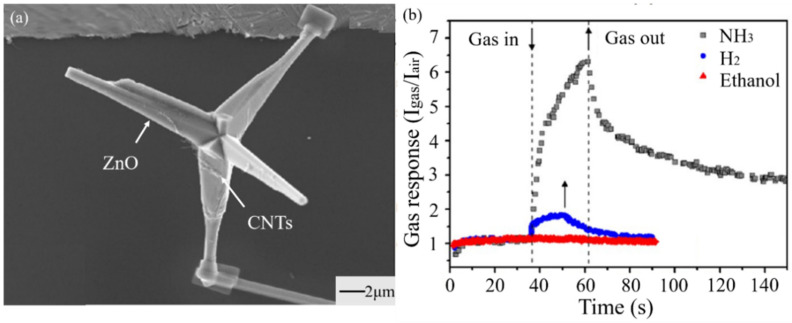
ZnO-T-CNT gas sensor. Adapted from [132] copyright (2017), with permission from American Chemical Society. (**a**) The morphology of CNTs and ZnO-T composite nanostructure; (**b**) the response of CNTs and ZnO-T composite nanostructure to NH_3_.

Carbon nanotubes and their products have excellent electrical conductivity and mechanical properties, but they cannot detect specific gases [131]. However, the composites of carbon nanotubes with metal and metal oxide not only inherit the unique properties of carbon nanotubes but also gain the ability to recognize some gases. Schutt et al. [132] obtained a mixed sensing material (ZnO-T-CNT) by attaching carbon nanotubes to the surface of a ZnO-T (tetrapodal ZnO) network, which had a high response to NH_3_ (Figure 9). ZnO-T-CNT had a porosity of up to 93%, which greatly enhanced the adsorption and desorption capacity of the mixed materials. Due to CNTs’ high conductivity, electrons can effectively transfer from ammonia molecules attached by CNTs to ZnO-T, enhancing the sensing performance of the network effectively. Bhat et al. [133] synthesized a ZnO-MWCNT nanocomposite. Although the sensitivity of the composite was less than that of pure ZnO, the response–recovery property was improved. In addition to the heterojunction interface between ZnO-MWCNT, another possible reason for the property improvement is MWCNTs acting as a catalyst in the experimental process. This leads to the change in the reaction rate by influencing various reaction sites on the surface and accelerates the overall response.

In summary, this method can effectively decrease the operation temperature and increase the selectivity of MOS gas sensors. The mechanism is mainly attributed to the increase in absorption sites and the formation of heterojunctions. More details about heterojunctions can be found in Section 3.4. This is also a factor, in that the carbon nanomaterials could effectively enlarge the channel of carrier transfer and accelerate the transfer of carriers to improve the properties [68]. Moreover, combining carbon nanomaterials with MOS nanomaterials can prove an effective method to improve the electron transfer in the interface. It provides an idea for the fabrication of heterojunction gas sensors. Additionally, owing to the fragile structure of carbon nanomaterials, we should prevent gas sensors from being destroyed in processing.

### 3.6. Conducting Polymers

**Figure 10:**
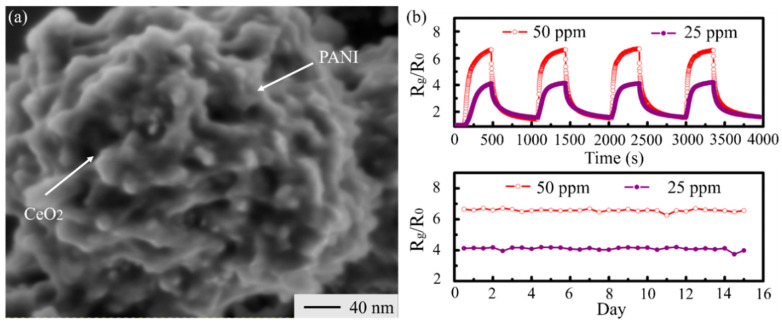
PANI-CeO_2_ gas sensor polymers. Adapted from [135] copyright (2014), with permission from American Chemical Society. [135]. (**a**) The morphology of PANI-CeO_2_ nanoparticles; (**b**) the response of PANI-CeO_2_ nanoparticles to NH_3_.

Due to conducting polymers’ sensing mechanism, their selectivity is higher than that of MOSs in general and the response is lower than that of MOSs [134]. Therefore, some researchers tried to use the conducting polymers and MOSs to overcome the shortcomings of MOS gas sensors. Wang and coworkers [135] synthetized a nanocomposite of polyaniline (PANI)-CeO_2_ C-S nanoparticles to detect NH_3_ (Figure 10). At room temperature, the response to 65 ppm of NH_3_ was 6.5. According to their experiment, this sensor can maintain this response for 15 days. Jiang et al. [136] also manufactured this nanocomposite of SnO_2_ and polypyrrole (Ppy) in their research, and it could detect 20 ppb of H_2_.

The sensing mechanism is characterized by the effect of heterojunctions and conducting polymers. The incorporation of conducting polymers with MOSs could increase the concentration of carriers and reaction sites, which is beneficial for the target gas adsorption. More details about heterojunction can be found in Section 3.4.

This method not only makes full use of the advantages of heterojunctions but also efficiently decreases the operation temperate of MOS gas sensors [135]. The disadvantage of this method is the high affinity of conductive polymers toward volatile organic compounds (VOCs) and humidity in the atmosphere [68]. It may be more suitable for making heterojunction sensors to detect inorganic gases. MOSs with conductive polymers could cause a response drop, which may increase the zero-drift of gas sensors. Therefore, we tried to use a function correction to decrease the zero-drift.

### 3.7. 2D Metal Dichalcogenides

**Figure 11:**
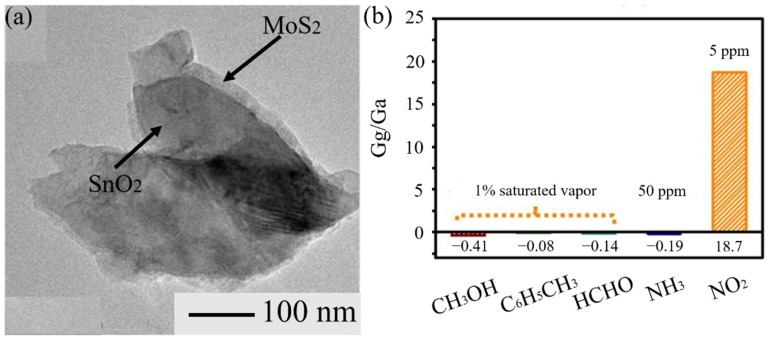
MoS_2_-SnO_2_ heterostructure gas sensor. Adapted from [137] copyright (2019), with permission from Elsevier. (**a**) The morphology of MoS_2_-SnO_2_ heterostructure; (**b**) the response of MoS_2_-SnO_2_ heterostructure to NO_2_.

Inspired by the appealing properties of graphene, researchers have made great efforts in exploring other 2D nanomaterials for gas sensing such as 2D metal dichalcogenides [17]. Han et al. [137] made a MoS_2_-SnO_2_ heterostructure gas sensor. It was able to detect NO_2_ at room temperature, and the response to 5 ppm of NO_2_ was 18.7. Compared with other gases, the selectivity was increased (Figure 11). Furthermore, this sensor had reliable long-term stability. Kim et al. [138] synthetized WS_2_-SnO_2_ C-S nanosheets by ALD. At the optimum shell thickness, this sensor indicated a good selectivity to CO. MoSe_2_ was used to manufacture the gas sensor. Abun et al. [139] designed a MoSe_2_-ZnO heterostructure gas sensor to detect H_2_. Compared with pristine ZnO and MoSe_2_, the selectivity and response were greatly increased.

The mechanism is due to the formation of heterojunctions. Under the action of heterojunction, response, selectivity, and operation temperature have been improved in varying degrees [137,138,139]. More details about heterojunctions can be found in Section 3.4. The combination of one-dimensional MOS material and two-dimensional metal material is a novel method owing to the large surface area and high surface-to-volume ratio. It is helpful to fabricate this type of heterojunction MOS gas sensor to detect inorganic gases. However, it is difficult to fit them firmly [46]. Therefore, it is necessary to improve the preparation process. Moreover, the unique layered structure of 2D metal dichalcogenides has provided the possibility of fabricating a flexible sensor substrate.

### 3.8. Temperature Modulating

**Figure 12:**
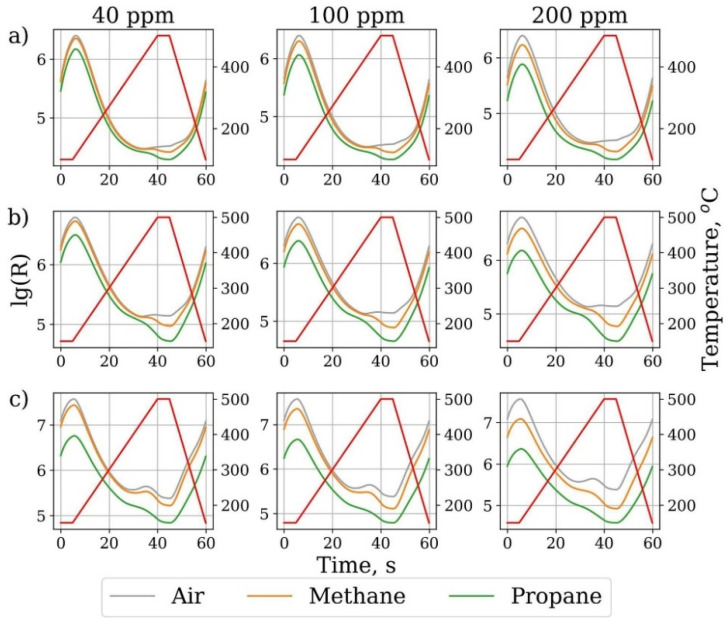
Schematic diagram of temperature modulation. Reprinted from [142] copyright (2021), with permission from Elsevier. (**a**) SnO_2_ sensors towards air, methane, and propane at different concentrations during a temperature modulation cycle; (**b**) Au-SnO_2_ sensors towards air, methane, and propane at different concentrations during a temperature modulation cycle; (**c**) Au/Pd-SnO_2_ sensors towards air, methane, and propane at different concentrations during a temperature modulation cycle.

Usually, gas sensors are used to monitor the target gas in a complex gas environment. There is the inevitable problem of cross-sensitivity. It could be understood that sensors have a response to multiple gases at the same time. Therefore, improving the selectivity of gas sensors is essential. Temperature modulating is considered a beneficial method to solve this problem. Yuan et al. [140] synthetized ZnO gas sensors detecting VOCs (volatile organic compounds) via the thermal method. To solve the cross-sensitivity, they used the trapezoidal wave temperature modulation improved by a rectangle to detect target gases and the GRNN to recognize gas species. The rates at which different gases combine with oxygen ions are different, so the optimal temperatures of reactions are different. With the trapezoidal change in temperature, the ZnO sensor showed a good response to every target gas at different temperatures in their experiment. Likewise, Yuan et al. [141] also discussed the feasibility of the application of temperature modulation to improve the rose-like MoO_3_/MoS_2_/rGO gas sensor selectivity to multiple gases (including acetone, methanol, ethanol, benzene, toluene, and ammonia). When exposed to the condition of temperature changing in the form of a sine wave, it showed the best response to ammonia and the lowest response to acetone. According to different responses to multiple gases, we could ensure it had a good selectivity to ammonia in multiple gases (including acetone, methanol, ethanol, benzene, toluene, and ammonia). Moreover, Krivetskiy et al. [142] proposed successfully using a Temperature modulation combined with statistical shape analysis to modify the SnO_2_/Au-SnO_2_/Au and Pd-SnO_2_ nanocrystalline gas sensor selectivity (Figure 12).

The mechanism can be summarized as follows: The reaction temperatures of different target gases and oxygen ions are different for a kind of nanomaterial-based gas sensor. When the ambient temperature changes with time for a certain range, each target gas has an optional reaction temperature at a certain point. Then, we can obtain a dynamic response curve, which can reveal the target gases and improve the selectivity of gas sensing (Figure 12). Based on the above, this method may provide a feasible way to detect both inorganic gases and VOCs. The advantage of this method is that it reduces the temperature drift effect by using the response information in static detection and eliminating the interference [141]. The category and concentration of mixed gases are distinguished by the errors in the distinction of adjacent concentrations [140]. Therefore, it is necessary to improve the recognition rate of the algorithm and the accuracy of the data processing algorithm. Moreover, the thermal stability of gas sensors should be improved to avoid their destruction by the temperature cycle.

### 3.9. Heating

**Figure 13:**
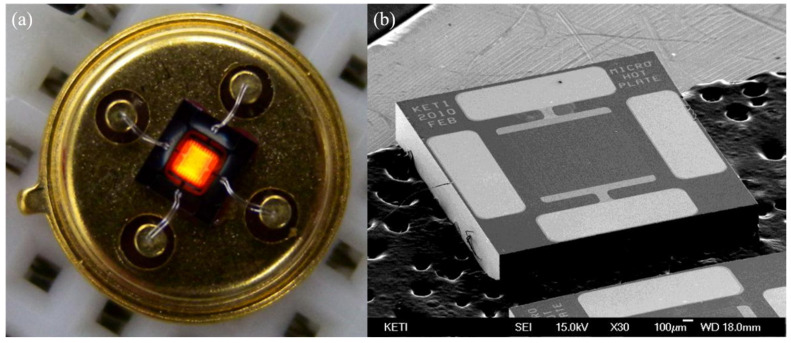
Schematic diagram of external micro-heater. Reprinted from [143]. (**a**) Packaged the micro-heater using TO39 package; (**b**) the SEM image of the micro-heater.

**Figure 14:**
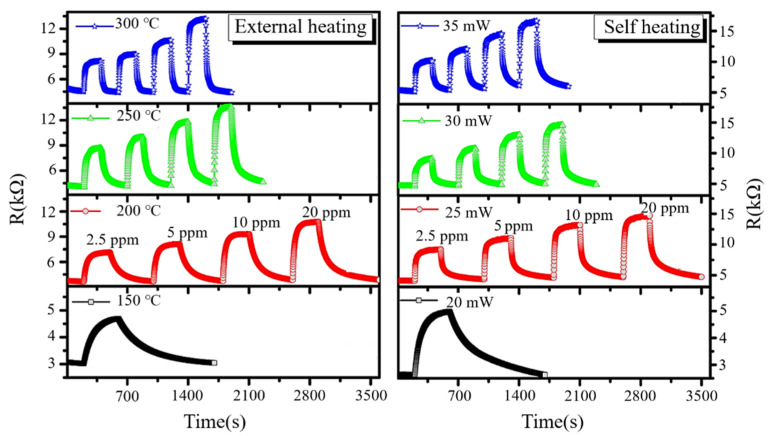
Performance of a sensor under external heating and self-heating effect. Adapted from [149] copyright (2017), with permission from American Chemical Society.

Heating is a method used to promote the property of gas sensors by assembling an extra heater [143,144,145,146] or the self-heating effect [147,148,149] to maintain the temperature condition that the gas reaction needs. An integrated heater is shown in Figure 13. Moon et al. [145] designed a NO_2_ gas sensor based on a microheater, which significantly reduced power consumption and recovery time and improved response. Compared with assembling an extra heater, the self-heating effect not only reduces the power consumption of the sensor but also facilitates the fabrication and miniaturization of sensor arrays. Tan et al. [149] designed a gas sensor based on SnO_2_ nanowires that can detect a NO_2_ concentration from 25.6 to 2.5 ppm, and they proved the feasibility of the self-heating effect (Figure 14). Moreover, they also demonstrated that the application of gas sensors with the self-heating effect could further reduce power consumption by narrowing the size of the sensor in the experiment. In general, the main function of heating is providing the operation temperature of the experiment and reducing humidity impact. Moreover, heating also contributes to the requirement of electron exchange between chemically adsorbed oxygen and MOS and between target gas and active oxygen species on the MOS surface [150]. Based on this information, we can infer that the electron is activated by heating to increase the possibility of electron transition.

As the gas reaction temperature is several hundred degrees Celsius, heating is an essential method for the improvement of gas sensor properties. However, the higher temperature could cause a decline in MOS gas sensors’ reliability. For example, with a reduction in the characteristic size of nanostructures, their melting temperatures decreased significantly [46]. Nanostructures in MOS sensors may be destroyed in a high-temperature environment. This is obviously harmful for their long-term application. Microheaters increase the volume of the whole sensor, and the circuit’s thermal consumption is inevitable [144,145]. If we integrate two kinds of gas sensors whose operation temperatures are similar, using an extra heater to provide a suitable temperature condition may be a good choice. Self-heating is helpful for the integration of other components and provides a new idea for wearable gas sensors [147,148,149]. Compared with extra heaters, the actual heating effect is difficult to control. We must consider the thermal loss by the substrate, the surrounding gases, and the contact pads [146]. Moreover, exploring a suitable MOS material to use in self-heating is also important. In the long term, both an extra heater and self-heating provide a suitable operation temperature to detect gas, which is harmful to the thermal stability of MOS gas sensors. Therefore, UV irradiation is a potential method for MOS gas sensing.

### 3.10. UV Irradiation

**Figure 15:**
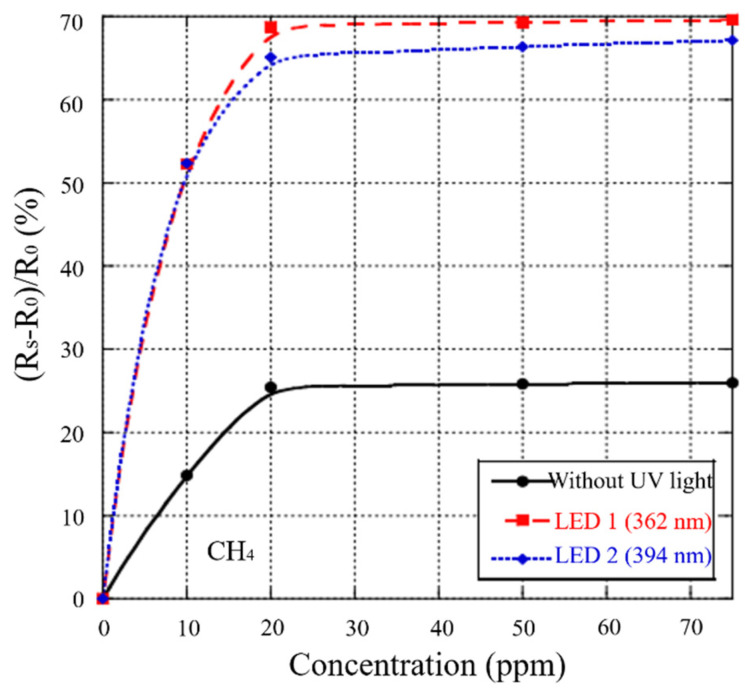
Performance of a gas sensor in the absence and presence of UV irradiation. Reprinted from [154] copyright (2016), with permission from Elsevier.

Although ultraviolet irradiation (UV irradiation) can also improve the performance from the perspective of energy, different from temperature modulation and heating, UV irradiation makes more use of photocatalysis, while the former is the change of thermal energy. To replace conventional high-temperature gas sensors, UV-LED can be used to enhance MOS gas sensors [151]. Karaduman et al. [152] manufactured NO_2_ gas sensors based on Al-Al_2_O_3_-p-Si and Al-TiO_2_-Al_2_O_3_-p-Si. The sensor response of two different materials was analyzed in a UV contrast test. The results showed that UV light can significantly improve the selectivity of a TiO_2_ sensor, and the response and recovery times were shortened to 6 and 12 s. Fan et al. [153] studied the properties of sensors based on zinc oxide film and nanowires in hydrogen; both sensors were able to detect hydrogen at the ppm level. The response of film and nanowire sensors with a width of 400 nm was increased to 9% and 19%, respectively (as shown in Figure 15). This method is beneficial for improving the response of MOS gas sensors [154]. Under the effect of UV irradiation, MOS material could absorb more energy, generate more charge carriers, and increase the density of free electron–hole pairs. It is helpful in promoting the electrical conductivity and properties of the sensor.

UV irradiation may be beneficial to achieve room-temperature gas sensing. It can effectively avoid high consumption and decrease thermal deformation by photocatalysis [155]. Moreover, UV irradiation also eliminates the influence of humidity. Water molecules are dissociated by photogenerated electrons and holes under different UV light intensities [156]. However, the disadvantages should not be ignored. For example, conventional UV lamps usually are large and make it difficult to integrate other sensors. The selectivity for reducing gases and VOCs has not obviously increased [155]. This is due to the interference of oxygen and other gases. Therefore, we can try to manufacture UV-LEDs and integrate them with MOS gas sensors to decrease the volume of the whole device, as well as decorating MOS gas sensors with some noble metals (Au, Ag). With the effect of noble metal, the selectivity will be improved. The synergistic effect of noble metal and UV irradiation is helpful to achieve gas sensing at room temperature. According to Trawka et al. [154], these gas sensing results mainly depend on the wavelength of ultraviolet light. Thus, determining how to precisely control the wavelength of ultraviolet light is a significant problem.

## 4. Conclusions

In this review, we summarized the advantages and disadvantages of MOS nanomaterials and introduced the main gas-sensing properties of MOS gas sensors. Then, we focused on the fabrication methods of interface micro–nanostructures to improve the gas-sensing properties.

MOS nanomaterials have become important nanomaterials in the fabrication of gas sensors owing to their high sensitivity, easy operation, and low cost. We can change the morphology of nanostructures by enlarging the surface-to-volume ratio in order to improve the gas-sensing properties. C-S nanostructures can improve the electron orientation through the formation of heterojunctions and the modulation of energy barriers. Carbon materials, conducting polymers, and 2D metal dichalcogenides improve the gas-sensing properties by forming heterojunctions. The carbon materials and conducting polymers increase the gas adsorption sites, while 2D metal dichalcogenides enlarge the surface-to-volume ratio. Although temperature modulation, self-heating effects, and UV irradiation methods can effectively enhance the MOS gas-sensing properties, the main limitations of the above methods mainly concern two aspects: On the one hand, MOS nanostructures are more fragile than conventional structures. MOS nanostructures are easily destroyed in the preparation of MOS gas sensors. On the other hand, MOS gas sensors can be deformed by thermal stress during the gas reaction.

In summary, thermal stability and structural stability are two important improvement directions for MOS gas sensors in the future. Additionally, UV irradiation provides a novel way to achieve room-temperature gas sensing. Thus, the C-S nanostructure can improve structural stability. These methods could provide some ideas to improve gas-sensing properties and point out development directions for MOS gas sensors. The advances in knowledge in all our endeavors can be a foundation and useful experience for sensing technology, surface science, catalysis, fluidic mechanics, and microelectronics.

## Figures and Tables

**Table 1:** Nanostructures decorated and loaded with noble metal catalysts.

Noble Metal Catalysts	Technique	Content	Material Structure	Operation Temperature (°C)	Target Gas	Gas Concentration (ppm)	Response (Ra/Rg)	Reference
Pd	Decorating	0.5 mol%	SnO_2_ Films	150	NO	0.5	542.8	[48]
	Decorating	/	SnO_2_ Nanowires	300	H_2_	1	8.02	[50]
	Loading	10 wt%	Co_3_O_4_ Membranes	150	H_2_	100	2.95	[51]
	Loading	/	Fe_2_O_3_ Nanocubes	139	Acetone	100	25.7	[49]
Pt	Decorating	6 %	ZnO Nanosheets	240	CH_4_	50	63.45	[52]
	Decorating	0.5 mol%	SnO_2_ Films	300	CO	150	406.2	[48]
	Loading	0.5 wt%	WO_3_ Mesoporous	125	CO	100	10 ± 1	[53]
	Loading	2 wt%	WO_3_ Nanosheets	300	Acetone	1.5	5.1	[54]
Au	Decorating	4 wt%	SnO_2_ Nanosheets	260	Ethanol	100	70.2	[55]
	Decorating	10%	VO_2_ Nanowires	25	NO_2_	5	3.22	[56]
	Decorating	1.5 wt%	SnO_2_ Nanoflowers	120	CH_4_	100	4.973	[57]
Ag	Decorating	1 wt%	ZnO Nanorods	360	Ethanol	50	21.5	[58]
	Decorating	0.5%	WO_3_ Films	200	NO_2_	3	12.22	[59]
	Loading	0.5%	WO_3_ Mesoporous	75	NO_2_	1	44	[60]
Rh	Decorating	2 deposition cycles	WO_3_ Films	350	CH_4_	5	63.1	[61]

**Table 2:** The conductivity of different MOSs in different gas conditions.

Semiconductor Type	Majority Carrier	Target Gas	Conductivity Performance
n-type	Free Electron	Oxidizing Gas	Reduce
		Reducing Gas	Increase
p-type	Hole	Oxidizing Gas	Increase
		Reducing Gas	Reduce

**Table 3:** Summary of some MOS C-S nanostructure applications.

C-S Heterostructure	Shell Deposition Technique	Nanostructure	Operation Temperature (°C)	Target Gas	Gas Concentration (ppm)	Response (Ra/Rg)	Reference
CuO-SnO_2_	ALD	Nanowires	250	HCHO	50	2.42	[101]
SnO_2_-NiO	ALD	Nanowires	500	H_2_	500	114	[97]
α-Fe_2_O_3_-ZnO	ALD	Nanowires	250	H_2_S	5	5.98	[76]
WO_3_-SnO_2_	ALD	Nanosheets	200	NH_3_	15	1.55	[99]
Ag-TiO_2_	Sol–gel	Nanowires	240	NH_3_	100	9	[78]
SnO_2_-Fe_2_O_3_	Sol–gel	Nanoparticles	Room Temperature	2-methoxyethanol	100	2080	[75]
Au-In_2_O_3_	Hydrothermal	Nanoparticles	300	H_2_	100	34.4	[107]
TiO_2_-NiO	Hydrothermal	Nanorods	400	Acetone	200	9.81	[79]
WO_3_-SnO_2_	Hydrothermal	Nanosheets	260	Acetone	50	32.1	[106]
In_2_O_3_-SnO_2_	Coaxial Electrospinning	Nanofibers	280	TMA	10	7.11	[102]
ZnO@In_2_O_3_	Coaxial Electrospinning	Nanofibers	225	Ethanol	100	31.87	[83]
Co_3_O_4_-α-Fe_2_O_3_	Coaxial Electrospinning	Nanofibers	240	Acetone	50	11.7	[103]
ZnO-CeO_2_	Coaxial Electrospinning	Nanofibers	370	Acetone	1	8.2	[90]

**Table 4:** Variation of depletion layer thickness in oxidizing gas and reducing gas.

Heterojunction Type	Main Carrier	Target Gas	Main Depletion Layer Types	Layer Thickness
p–n Type	Free Electrons and Holes	Oxidizing Gas	Electron Depletion Layer	Increase
			Hole Depletion Layer	Reduce
		Reducing Gas	Electron Depletion Layer	Reduce
			Hole Depletion Layer	Increase
n–n Type	Free Electrons	Oxidizing Gas	Electron Depletion Layer	Increase
		Reducing Gas		Reduce
p–p Type	Holes	Oxidizing Gas	Hole Depletion Layer	Reduce
		Reducing Gas		Increase

## Data Availability

No new data were created in this study.
